# Hedgehog stimulates hair follicle neogenesis by creating inductive dermis during murine skin wound healing

**DOI:** 10.1038/s41467-018-07142-9

**Published:** 2018-11-21

**Authors:** Chae Ho Lim, Qi Sun, Karan Ratti, Soung-Hoon Lee, Ying Zheng, Makoto Takeo, Wendy Lee, Piul Rabbani, Maksim V. Plikus, Jason E. Cain, David H. Wang, D. Neil Watkins, Sarah Millar, M. Mark Taketo, Peggy Myung, George Cotsarelis, Mayumi Ito

**Affiliations:** 1 The Ronald O. Perelman Department of Dermatology and Department of Cell Biology, New York University School of Medicine, New York, NY 10016 USA; 2 Department of Dermatology and Cell and Developmental Biology, Perelman School of Medicine, University of Pennsylvania, Philadelphia, PA 19104 USA; 3 Department of Developmental and Cell Biology, Irvine, Sue and Bill Gross Stem Cell Center, University of California, Irvine, CA 92617 USA; 4 Centre for Cancer Research, Hudson Institute for Medical Research and Department of Molecular and Translational Science, Faculty of Medicine, Nursing and Health Sciences, Monash University, Clayton, VIC 3816 Australia; 5 Division of Hematology and Oncology, Department of Internal Medicine, Harold C. Simmons Comprehensive Cancer Center, Esophageal Diseases Center, Medical Service, VA North Texas Health Care System, University of Texas Southwestern Medical Center, 5323 Harry Hines Boulevard, Dallas, TX 75390 USA; 6 The Kinghorn Cancer Centre, Garvan Institute of Medical Research, Darlinghurst, NSW 2010 Australia; 7 Division of Experimental Therapeutics, Graduate School of Medicine, Kyoto University, Kyoto, 606-8501 Japan; 8 Departments of Dermatology and Pathology, School of Medicine, Yale University, New Haven, CT 06520 USA

## Abstract

Mammalian wounds typically heal by fibrotic repair without hair follicle (HF) regeneration. Fibrosis and regeneration are currently considered the opposite end of wound healing. This study sought to determine if scar could be remodeled to promote healing with HF regeneration. Here, we identify that activation of the Sonic hedgehog (Shh) pathway reinstalls a regenerative dermal niche, called dermal papilla, which is required and sufficient for HF neogenesis (HFN). Epidermal Shh overexpression or constitutive Smoothened dermal activation results in extensive HFN in wounds that otherwise end in scarring. While long-term Wnt activation is associated with fibrosis, Shh signal activation in Wnt active cells promotes the dermal papilla fate in scarring wounds. These studies demonstrate that mechanisms of scarring and regeneration are not distant from one another and that wound repair can be redirected to promote regeneration following injury by modifying a key dermal signal.

## Abstract (web summary)

On wounding, scar formation in mammals arises causing no hair follicle regeneration, but it is unclear if scarring precludes regeneration. Here, the authors show that if Sonic hedgehog signaling is activated in the wound, an inductive dermal niche forms, enabling regeneration and hair follicle formation.

## Introduction

The hair follicle (HF) is a complex mini-organ that is formed during embryonic development through communication between follicular epithelial cells and underlying dermal papilla cells (DP)^1,2^. DP cells constitute the dermal niche that instructs hair follicle epithelial cell fate and differentiation^3,4^. After embryonic follicular development, interactions between HF resident stem cells (HFSCs) and the underlying DP continue to regulate the cyclical regeneration of hair shafts in existing HFs throughout the life of the organism^5–7^. However, the de novo generation of HFs is rare in adult animals. Failure to regenerate HFs that are lost from injury or disease represents a major challenge in cutaneous regenerative medicine^8,9^ and can be largely traced to the resistance of adult mammals to resurrect complex embryonic epithelial–mesenchymal interactions that govern organogenesis.

Most adult mammalian tissues do not undergo regenerative healing, a process that recreates a functional tissue nearly indistinguishable from its original form^10^. However, healing of large skin excisions in mice has been shown to recapitulate aspects of true tissue regeneration in the formation of new HFs (HF neogenesis, HFN) thus providing a rare example of mammalian epimorphic regeneration^11–13^. In contrast, small skin excisions in mice result in the formation of scar tissue, or fibrosis, which is composed largely of extracellular matrix components like collagens and fibronectin and devoid of new accessory structures like HFs^11^ (Supplementary Fig. 1a–g). Both large and small human skin wounds incurred by trauma or dystrophy almost always undergo fibrotic scarring.

As yet, the mediators that determine the balance between scar-forming and regenerative skin wound healing are unknown. The relationship between scarring and regeneration is currently elusive. It is unknown whether scarring precludes regeneration, whether scarring cells intrinsically lack the competence for regeneration or whether regenerative cues are simply absent. In this study, we reveal that Shh signaling plays a major role in converting wound fibroblasts from scar promoting, to those that stimulate HFN. Our study suggests that the absence of regenerative cues impacts the inability of hair follicle morphogenesis after injuring the skin.

## Results

### Shh signaling is essential for wound-induced HF neogenesis

**Fig. 1:**
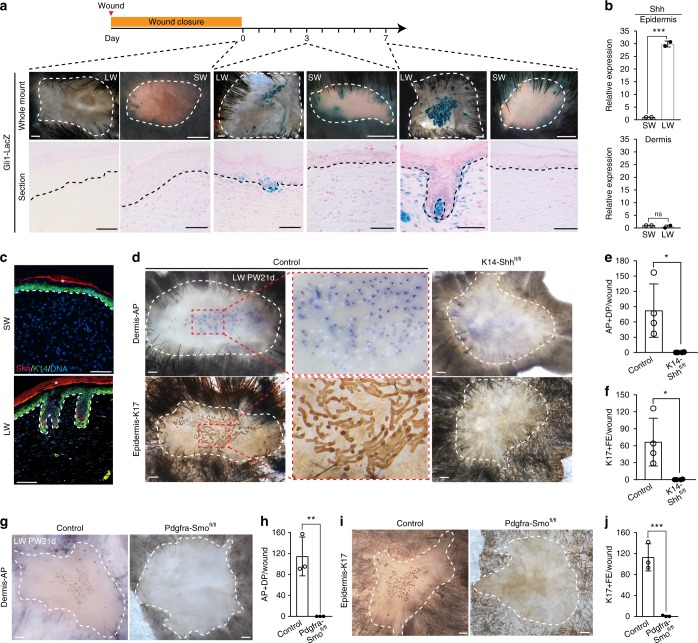
Shh signaling is necessary for HFN following injury. **a** X-gal staining of large wound (LW) and small wound (SW) from of *Gli1-LacZ* mice at indicated time (*n* = 2–4 W (2–5 M) per condition). **b** qRT-PCR for Shh expression in SW and LW of wild-type mice at 7 days after complete re-epithelialization (*n* = 2–6 W (2 M) per condition). **c** Immunohistochemistry of Shh on SW and LW of wild-type mice at 7 days after complete re-epithelialization. *Indicates non-specific signals. **d**–**f** *K14-CreER; Shh* ^*fl/fl*^ (*K14- Shh* ^*fl/fl*^) and littermate control mice were subjected to large wound (LW) and treated with TAM from PW3d until tissue harvest at PW21d (*n* = 11–12 W (11–12 M) per condition). Whole-mount HFN assay (**d**) and quantifications (**e**, **f**). **g**–**j** *Pdgfra-CreER; Smo* ^*fl/fl*^ (*Pdgfra-Smo* ^*fl/fl*^) and littermate controls were subjected to large wound (LW) and were treated with TAM from 3 or 4 weeks before wounding until tissue harvest at PW21d (*n* = 3–5 W (3–5 M) per condition). Whole-mount HFN assay (**g**, **i**) and quantifications (**h**, **j**). *n*: number of wounds (W) or mice (M), Data are represented as mean ± s.d., *p < 0.05; **p < 0.01; ***p < 0.001; Student’s *t*-test, Dashed white circle: wound boundary, dashed line: epidermis–dermis border, DP dermal papilla, AP alkaline phosphatase, FE follicular epithelium, PW post-wound, Scale bars represent 500 µm (**a** (whole mount), **d**, **g**, **i**), 50 µm (**a** (section), **c**)

In analyzing the disparate healing responses in large and small wounds in adult mice, we found that Gli1 expression, a readout of Hh pathway activation, was localized to the center of large wounds, corresponding to regions of hair placode/germ formation. In striking contrast, it was absent from small wounds (Fig. 1a and Supplementary Fig. 1h). The Gli1 signal in large wounds localized to both DP and epithelial hair germ cells, recapitulating the pattern observed in embryonic HF development^14^. Moreover, Shh, a major ligand of the pathway, was upregulated at the site of HFN in the epithelial compartment in large wounds but absent from either epidermal or dermal compartment in small wounds (Fig. 1b, c).

Shh expression during hair follicle morphogenesis is conserved between mice and humans^15^ and vital for hair follicle development and hair cycle^14,16–22^. We asked if the absence of Hh pathway activation in mouse small wounds might explain their failure to undergo regenerative wound healing during wound repair.

First, to understand the importance of epithelial Shh expression in large wounds, *Shh* was genetically deleted from epidermal cells in healed large wounds of *K14-CreER; Shh* ^*fl/fl*^ mice upon tamoxifen (TAM) induction from post wound (PW)3d to PW21d. This resulted in a loss of DP and hair germ formation compared to control mice (Fig. 1d–f). These results showed that epithelial Shh ligands are essential for DP formation and HFN. Additionally, deletion of Smo, an essential component of Shh pathway activation, in underlying wound dermal cells in TAM-treated *Pdgfra-CreER; Smo* ^*fl/fl*^ *mice* also resulted in inhibition of DP formation and associated HFN events (Fig. 1g–j). Thus, activation of the Shh signaling pathway in the wound dermis plays a vital role in promoting DP formation.

### Epithelial Shh leads to HF neogenesis in wounds

**Fig. 2:**
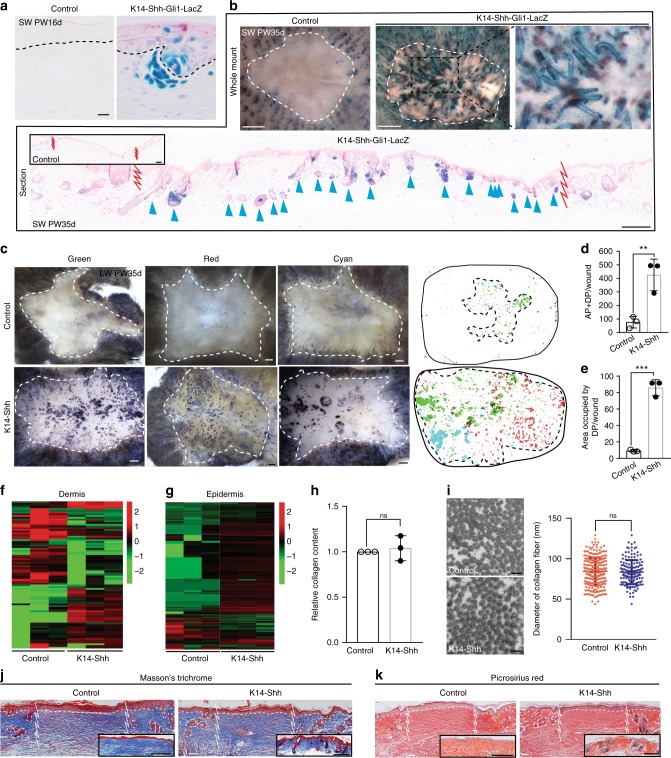
Epidermal Shh is capable of regenerating HF in wounds without alteration of collagen. **a**, **b** *K14-CreER; LSL-Shh*; *Gli1-LacZ* (*K14-Shh-Gli1-LacZ*) and littermate controls with *Gli1-LacZ* were subjected to SW and treated with TAM from PW1d until tissue harvest at indicated time (*n* = 12 W (4 M) per condition). X-gal staining was analyzed at PW16d (**a**), and PW35d (**b**). Arrowheads show regenerated HFs (**b**). **c**–**e** Distribution of regenerated DP was analyzed from three representative LWs of *K14-CreER; LSL-Shh* (*K14-Shh*) and littermate controls treated with TAM from PW3d until tissue harvesting at PW35d (*n* = 9 W (9 M) per condition). AP^+^ DP in each picture were converted into dots with three different colors (red, green, and cyan) and merged into one picture (**c**, right). The original images and the corresponding colors represented in three columns on the left (**c**). Quantifications of AP^+^ DP per wound (**d**) and area occupied by DP (**e**). “Area occupied by DP” was defined by drawing a line that connects the outermost regenerated DP in wound. **f**–**k** *K14-CreER; LSL-Shh* (*K14-Shh*) and littermate controls were subjected to SW and treated with TAM from PW1d until tissue harvest at indicated time. RNA-seq analyses at PW11d showing heatmap of differentially expressed genes for *K14-Shh* and control mice (**f**, **g**). Red and green correspond to high and low expression levels, respectively (*n* = 12 W (4 M) per condition). Hydroxyproline assay to measure collagen contents at PW11d (*n* = 12 W (4 M) per condition) (**h**). Diameter of collagen fiber at PW30d (TEM images on the left, *n* = 2 W (2 M) per condition) (**i**). Detection of collagen fiber by Masson’s trichrome staining (**j**) and Picrosirius red staining (**k**) at PW11d and PW35d (insets). *n*: number of wounds (W) or mice (M), Data are represented as mean ± s.d., **p < 0.01; ***p < 0.001; ns: non-significant; Student’s *t*-test, Zigzag line and dashed white circle: wound boundary, Dashed line: epidermis–dermis border, SW small wound, LW large wound, PW post-wound, DP dermal papilla, AP alkaline phosphatase, FE follicular epithelium. Scale bars represent 500 μm (**b** (whole mount), **c**), 50 μm (**b** (section), **j**, **k**), 10 μm (**a**), 100 nm (**i**)

To determine if Shh activation could induce HFN in scar-forming wounds, we overexpressed Shh in epithelial cells in *K14-CreER; LSL-Shh* or *K14-CreER; LSL-Shh; Gli1-LacZ* mice and examined HFN in small wounds^23^. To induce Shh overexpression, TAM was injected into control and *K14-CreER; LSL-Shh* or *K14-CreER; LSL-Shh; Gli1-LacZ* mice from PW1d to indicated time points in Fig. 2. This treatment resulted in extensive HFN in wounds compared to control wounds (Fig. 2a, b). Epidermal Shh overexpression resulted in Gli1 activation in both the epidermis and dermis, corresponding to the areas of HFN. Shh-driven hair germs expressed Lef1 and K17 and exhibited normal hair follicle morphogenesis (Supplementary Fig. 2a–c, f–i). AP^+^ DPs were associated with overlying K17^+^ epithelial buds as typically observed in HFN (Supplementary Fig. 2d)^11^. Eventually, many of these hair follicles (52 ± 16%, mean ± s.d.) grew downward to form mature hair follicles with hair shafts, an event rarely observed in control small wounds (Supplementary Fig. 2e). New DP expressed Lef1 and Noggin as well as AP, further demonstrating their DP identity **(**Supplementary Fig. 2f**)**^24,25^. We rarely found aberrant basaloid growths resembling superficial basal-cell carcinomas (BCCs) in these wounds.

Previous studies noted that HFN in large wounds in WT mice was limited to the central area of the wounds^11^. Given the ability of Shh to induce ectopic HFN in small wounds, we asked if exogenous Shh might overcome the inability of new hair follicles to form outside this central region in large wounds. In large wounds from TAM-treated *K14-CreER; LSL-Shh* mice, we observed extensive DP formation covering the entire wound area compared with TAM-treated controls. **(**Fig. 2c–e**)**. These results verify the potency of Shh activation to overcome regional inhibition of HFN.

To characterize changes in gene expression following Shh overexpression, TAM was administered into control and *K14-CreER; LSL-Shh* mice from PW1d to PW11d and wound cells isolated for RNA-seq analyses. Epidermal and dermal cells from TAM-treated control and *K14-CreER; LSL-Shh* mice were compared by RNA-seq analyses **(**Fig. 2f, g and Table 1 and Supplementary Fig. 3**)**. Gene ontology (GO) analyses showed that processes and signatures involved in embryonic HF morphogenesis, including cell proliferation, cell adhesion, and Hh signaling^1^, were enriched by Shh overexpression **(**Supplementary Fig. 3a, c**)**. There was no significant difference in expression of *Fgf9*, which is known to promote hair follicle neogenesis in a large wound model, either in the epidermis (FDR:0.64) or dermis (FDR:0.97) of *K14-CreER; LSL-Shh* mice compared to controls. Most notably, there was upregulation of the Shh signaling pathway in both the epithelial cells and dermal cells. We also noted upregulation of DP signature genes *Bmp7*, *Enpp2*, *lamc3*, and *Trps1* in the wound dermis and hair placode signature genes, including *Trp73, Vwa2, Samd5, Cxcl14, Nedd9, and Tnfaip3* in the wound epidermis of Shh overexpressed mice (Supplementary Fig. 3b, d)^26^. These data were confirmed by qPCR analyses (Supplementary Fig. 3e, f**)**. These results suggest that epidermal Shh overexpression can induce key embryonic signatures of HF morphogenesis.

**Table 1:** Comparison of Hh pathway component and collagen expression between *K14-Shh* and control mice based on RNA-seq

		Genes	Fold change	FDR
Dermis	Hh pathway	*Shh*	2.41	0.64
		*Gli1*	3.20	0.16
		*Gli2*	4.18	0.06
		*Ptch1*	3.96	0.03
	Collagen	*Col1a1*	0.95	0.99
		*Col1a2*	1.00	0.99
		*Col3a1*	0.94	0.99
Epidermis	Hh pathway	*Shh*	51.83	2.01E-04
		*Gli1*	4.21	1.33E-01
		*Gli2*	7.27	8.65E-07
		*Ptch1*	3.74	3.08E-04
	Collagen	*Col1a1*	0.56	0.56
		*Col1a2*	0.82	0.87
		*Col3a1*	0.86	0.91

### Epithelial Shh regenerates HFs without altering neighboring collagen

Although the increased collagen I deposition by adult dermal fibroblasts vs. fetal fibroblasts during wound healing is well-established, whether the low level of collagen I is essential for embryonic/neonatal non-scarring healing is currently unknown. Our RNA-seq suggests that epithelial Shh overexpression did not significantly change the overall extracellular matrix composition of the wound dermis towards an embryonic state. The increased ratio of type III versus type I collagen is a key characteristic of fetal scarless wound healing^27–30^, and expressions of the genes encoding these collagens were unchanged (FDR: 0.99 for *Col1a1, Col1a2*, and *Col3a1*) (Table 1). This was verified by biochemical measurement for the content of hydroxyproline, a major component of collagen (Fig. 2h). In addition, transmission electron microscopy (TEM) analysis of the wound area showed no change in collagen fiber diameter due to Shh overexpression, which is directly proportional to the tensile strength^31^ (Fig. 2i). Consistently, no significant differences were noted in the histological assessments for collagen staining (i.e., Masson trichrome and Picrosirius red staining) before and after the formation of neogenic hair follicles (Fig. 2j, k). We found that the epithelial Shh expression and the reduction of type I collagen in the neighboring wound scar does not play a significant role in Shh-driven HF neogenesis. The data suggests that DP and hair follicles were forming within the scar of the wounds.

### Dermal Hh activation induces HF neogenesis in scarring wounds

**Fig. 3:**
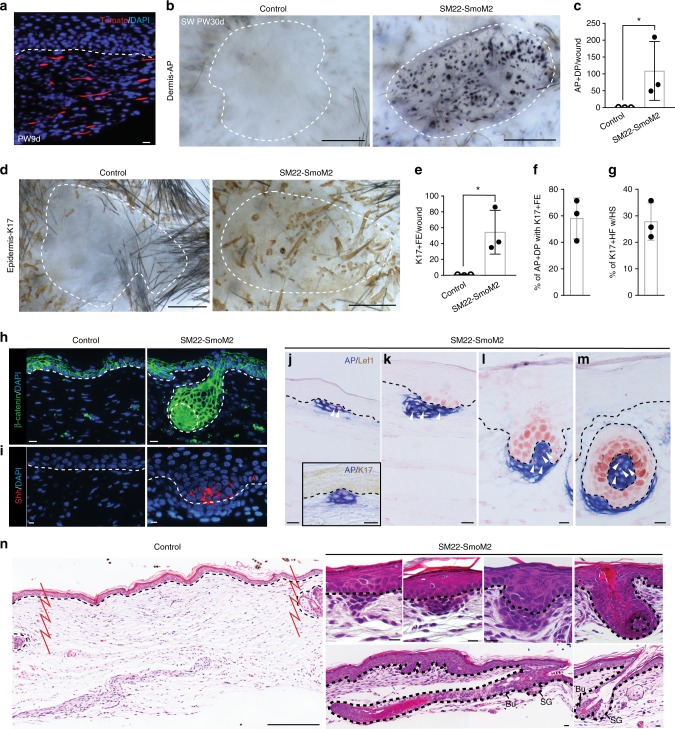
Dermal Hh activation is sufficient to promote HFN in non-regenerative wounds. **a** Detection of tomato reporter on SW of *SM22-rtTA; tetO-Cre; R26-Tomato* mice at indicated time. **b**–**n** *SM22-rtTA; tetO-Cre: R26-SmoM2* (*SM22-SmoM2*) and littermate controls were subjected to SW and treated with doxycycline from PW1d until tissue harvest at PW30d (*n* = 18 W (4–5 M) per condition). Whole-mount HFN assay (**b**, **d**) and quantifications (**c**, **e**). Percentage of AP^+^ DP with K17^+^ FE (**f**). Percentage of K17^+^ HF with hair shaft (HS) (**g**). Immunohistochemistry with indicated markers (**h**–**m**) and H&E (**n**). *n*: number of wounds (W) or mice (M), Data are represented as mean ± s.d., *p < 0.05; Student’s *t*-test, Dashed white circle: wound boundary, Dashed line: epidermis–dermis border, SW small wound, PW post-wound, DP dermal papilla, AP alkaline phosphatase, FE follicular epithelium, HF hair follicle, HS hair shaft, Bu bulge stem cell area, SG sebaceous gland. Scale bars represent 500 µm (**b**, **d**), 100 µm (**n** (Control)), 10 µm (**a**, **h**–**m, n** (SM22-SmoM2))

To ask if direct Shh pathway activation of myofibroblasts in the small wound dermis would also promote DP formation and HFN, we induced expression of the activated form of Smo under the control of the *SM22α* promoter, known to be specifically active only in dermal myofibroblasts (*SM22-rtTA; tetO-Cre; R26-SmoM2*) (Fig. 3a)^32–34^. In the transgenic mice, constitutively active Smo expression is dependent on doxycycline (dox) administration. Expression of the activated form of Smo in myofibroblasts during wounding (dox treatment from PW1d to PW30d) or following re-epithelialization (dox treatment from PW10 ± 2d to PW46d) resulted in DP formation within small wounds of *SM22-SmoM2* mice compared to control mice **(**Fig. 3b, c and Supplementary Fig. 4a, b**)**. Constitutive Hh activation in wound myofibroblasts from *SM22-SmoM2* mice also resulted in striking changes in wound epithelial cells. Those epithelial cells directly above Smo-active DP expressed K17 and Shh, well-established hair germ markers (Fig. 3d, e, i and Supplementary Fig. 4a, c, 6a). Additionally, these hair germ cells displayed nuclear β-catenin and Lef1 expression indicating active Wnt signaling, a known signature of hair follicle development and growth (Fig. 3h, j-m)^11,35^. A significant number of new hair follicles were also observed with differentiated hair shafts (28±7%) containing both outer root sheath (AE13^+^ hair cortex, AE15^+^ medulla)^36,37^, and inner root sheath (AE15^+^)^37^ structures, stem cell compartments (K15^+^CD34^+^)^38,39^, hair matrix cells (Shh^+^) (Fig. 3g, n and Supplementary Fig. 5). Neogenic hair follicles also maintained an adjoining dermal sheath (SMA^+^, SM22α^+^)^40^, biochemically distinct from DP (Noggin^+^)^25^ (Supplementary Fig. 5). We also frequently observed AP^+^ DP structures without accompanying hair germs despite their close proximity to overlying epidermis (Fig. 3j). Indeed, there were almost twice as many AP^+^ DP as K17^+^ hair germs, suggesting that many DP (42±15%) formed without establishing epithelial–mesenchymal interactions that could promote HF morphogenesis (Fig. 3f). DP formation without hair germ formation is not observed in normal endogenous hair follicle development in embryo or in adult large wounds^11^. These regenerative events were observed following wound closure. Intriguingly, even in the experiments where we induced Hh activation early during wound healing, the time of wound closure, proliferation, epidermal differentiation, AP distribution, angiogenesis and infiltration of immune cells including macrophages into the wound site were not altered prior to wound closure (Supplementary Fig. 6b-i).

### Hh activation shifts dermal fibroblast fate toward DP

**Fig. 4:**
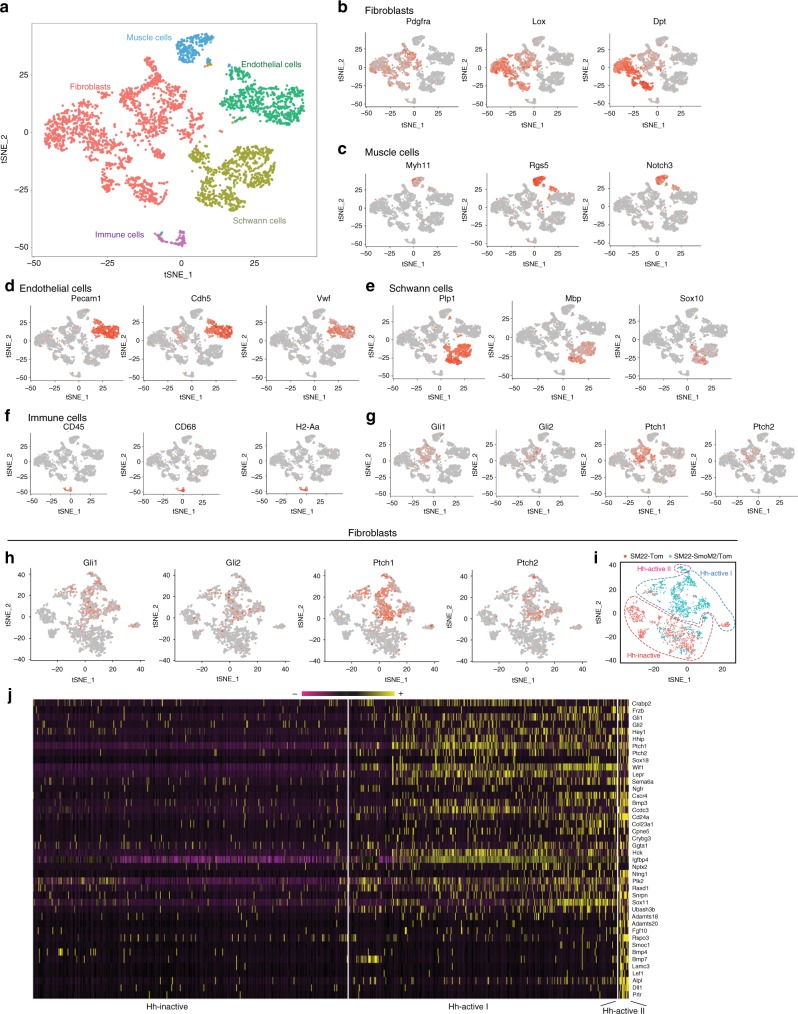
Dermal Hh activation induces DP fate in wound fibroblasts. scRNA-seq was performed with Tomato^+^ cells isolated from wound dermis of both *SM22-rtTA; tetO-Cre; R26-SmoM2/Tomato* (*SM22-SmoM2*) and *SM22-rtTA; tetO-Cre; R26-Tomato* (control) mice 3 days after complete re-epithelialization. The mice were subject to SW and treated with doxycycline from PW1d until PW12d (*n* = 12–25 W (3–5 M) per condition). **a**–**i** tSNE plots of 4680 SM22 + dermal cells split between control and Hh activated conditions. tSNE plot of SM22^+^ dermal cells colored by assigned lineages (**a**). tSNE plot of SM22^+^ dermal cells according to lineage-specific markers (**b**–**f**). tSNE plot of SM22^+^ dermal cells according to Hh pathway components (**g**). tSNE plot of SM22^+^ myofibroblasts according to Hh pathway components (**h**). tSNE plot of SM22^+^ myofibroblasts according to cell origin (**i**). **j** Heatmap showing the expression of DP signature genes. Yellow and black/purple correspond to high and low expression levels, respectively

To understand whether SM22^+^ dermal cells universally induce the DP fate upon Hh activation, we isolated Tomato^+^ cells from wound dermis of *SM22-rtTA; tetO-Cre; R26-SmoM2/Tomato* (*SM22-SmoM2*) and *SM22-rtTA; tetO-Cre; R26-Tomato* (control) mice 3 days after complete re-epithelialization (dox treatment from PW1d to PW12d) and compared their molecular signatures by single-cell RNA sequencing (scRNA-seq) (Fig. 4). We performed unsupervised clustering with K means based on differentially expressed genes (DEGs), using tSNE (t-distributed stochastic neighbor embedding). Based on expression of lineage markers for different cell types, we identified cellular clusters of fibroblasts, muscle cells, schwann cells, endothelial cells and immune cells (Fig. 4a–f)^41–44^. Examination of Hh pathway mediators *Gli1*, *Gli2*, *Ptch1*, and *Ptch2* showed that Hh pathway components were mainly expressed in the fibroblast cluster (Fig. 4g) and were largely restricted to the *SM22-SmoM2* group (Fig. 4h, i). This is consistent with the lack of *Gli1-lacZ* expression in the wild-type dermis of small wounds (Fig. 1a and Supplementary Fig. 1h). The Hh-active fibroblast clusters showed upregulation of DP signature genes including *Hey1*, *Sema6a*, *Wif1*, *Cxcr4*, *Ggta1*, *Hck*, *Snrpn* and *Rasd1* as previously defined by several groups (Fig. 4j)^26,45–48^, suggesting that Hh activation in SM22^+^ myofibroblasts are sufficient to globally induce upregulation of DP signature genes. Nonetheless, within this Hh-activated fibroblast population, we identified a divergence in the number and level of DP signature genes (Hh-active I and II), suggesting that Hh-independent mechanisms are also involved in the regulation of DP signature genes. For example, while the upregulation of *Bmp3* and *Plk2*, known DP signature genes, was widely observed among Hh-active fibroblasts (Hh-active I and II), expression of *Alpl* (*AP*) and *Lef1*, vital markers for DP identification in histochemical analyses, was limited to a small subpopulation of Hh-active fibroblasts (Hh-active II) (Fig. 4j).

### Hh activation in wound epidermis forms BCC-like structure

In contrast to dermis-specific activation of the Hh pathway, forced Hh activation solely in epithelial cells (*K14-CreER; R26-SmoM2*, TAM administration from PW1d to PW30d) did not promote DP formation in the wound area (Supplementary Fig. 7). As previously observed, the epidermis of these wounds maintained numerous K17^+^ epithelial invaginations resembling BCCs without signs of hair follicle differentiation^49–51^. Also, the Hh-driven, BCC-like epithelial growths were not accompanied by underlying DP^52^.

### Hh activation converts Wnt-active wound fibroblasts into DP

**Fig. 5:**
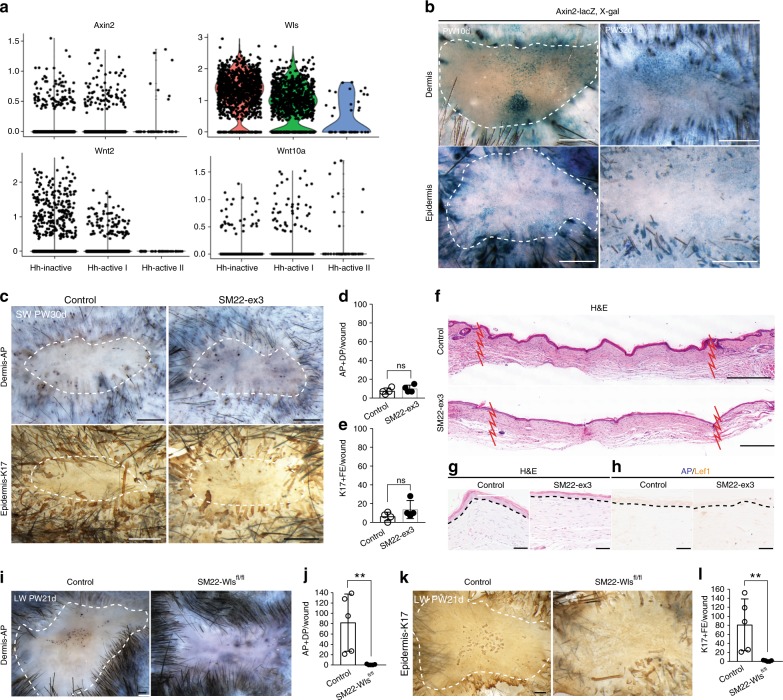
Dermal Wnt activation alone is not sufficient for HFN. **a** Violin plots of Wnt pathway-related genes in SM22^+^ myofibroblasts. **b** X-gal staining in epidermis and dermis of SW of *Axin2-LacZ* mice at PW10d and PW32d. **c**–**h** *SM22-rtTA; tetO-Cre; β-catenin* ^*fl(ex3)/+*^ (*SM22-ex3)* and littermate controls were subjected to SW and treated with doxycycline from PW1d until tissue harvest at PW30d (*n* = 3–4 W (3–4 M) per condition). Whole-mount HFN assay (**c**) and quantifications (**d**, **e**). H&E (**f**, **g**) and AP/Lef1 staining (**h**) show lack of hair germ (HG) formation by *β-catenin* activation in dermis. **i**–**l** *SM22-rtTA; tetO-Cre; Wls* ^*fl/fl*^ (*SM22-Wls* ^*fl/fl*^*)* and littermate controls were subjected to LW and treated with doxycycline from PW3d until tissue harvest at PW21d (*n* = 4–5 W (4–5 M) per condition). Whole-mount HFN assay (**i**, **k**) and quantifications (**j**, **l**). *n*: number of wounds (W) or mice (M), Dashed white circle and zigzag line: wound boundary, Dashed line: epidermis–dermis border, SW small wound, LW large wound, AP alkaline phosphatase, PW post-wound, Scale bars represent 500 µm (**b**, **c**, **i**, **k**), 100 µm (**f**), 50 µm (**g**, **h**)

Next, given that Wnt signaling plays an essential role in hair follicle development and neogenesis^11,53–56^, we sought to examine the relationship between Wnt and Shh signaling. Our scRNA-seq analyses in myofibroblasts and examination of Axin2 expression in wounds from *Axin2-LacZ* mice both showed Wnt activity in scarring dermis of small wounds. scRNA-seq analyses demonstrated the expression of *Axin2*, *Wls*, and canonical *Wnt* ligands such as *Wnt2* and *Wnt10a* (Fig. 5a, b). Our findings are consistent with previous reports that long-term Wnt signaling within dermal fibroblasts correlates with injury-induced fibrosis^57,58^. We hypothesized that dermal Wnt signaling is not sufficient to induce HFN without Hh activation. Indeed, constitutive activation of Wnt signaling (*SM22-rtTA; tetO-Cre; β-catenin* ^*ex3/fl*^) in SM22^+^ dermal cells of small wounds did not promote new DP regeneration or HFN (Fig. 5c–h). However, dermal depletion of Wls, which is essential for Wnt ligand secretion, inhibited HFN in large wounds (*SM22-rtTA; tetO-Cre; Wls* ^*fl/fl*^ and dox treatment from PW3d to PW21d). These results suggest the requirement of dermal Wnt ligands for HFN (Fig. 5i–l).

**Fig. 6:**
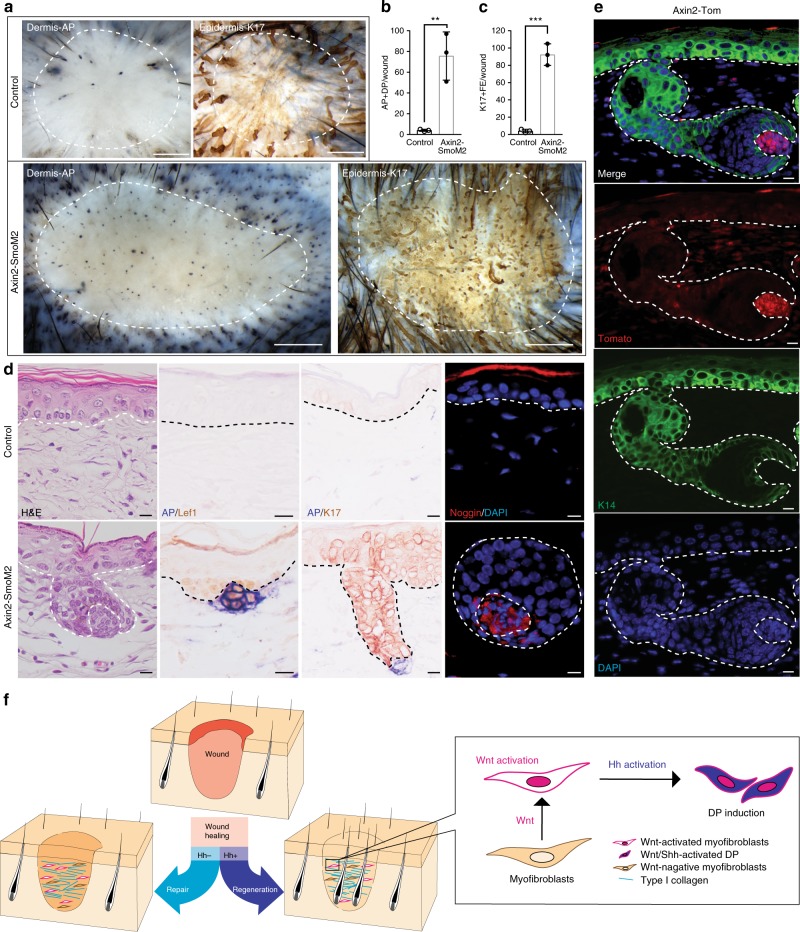
Hh activation can convert fibrotic Wnt-active dermal cells into DP in wounds. **a**–**d** *Axin2-CreER; R26-SmoM2 (Axin2-SmoM2)* and littermate controls were subjected to SW and treated with TAM from PW1d until tissue harvest at PW30d (*n* = 9–15 W (3–5 M) per condition). Whole-mount HFN assay (**a**) and quantifications (**b**, **c**). H&E and immunohistochemical analyses with indicated markers (**d**). **e** Tracing of tomato-labeled Axin2^+^ cells in *Axin2-CreER; R26-Tomato (Axin2-Tom). Axin2-Tom* mice were subjected to LW and treated with TAM from PW3d until PW12d (before complete re-epithelialization). Tissue was harvested at PW23d and stained with anti-RFP antibody. **f** Model: Conversion of wound repair to regeneration in adult skin. Wound healing in mammalian skin typically results in scarring and lack of appendage regeneration. Dermal Hh activation can install de novo dermal papilla into wounds, resulting in regenerative HF neogenesis, despite collagen deposition in adult wounds. *n*: number of wounds (W) or mice (M), Data are represented as mean ± s.d., **p < 0.01, ***p < 0.001; Student’s *t*-test, Dashed white circle: wound boundary, Dashed line: epidermis–dermis border, SW small wound, LW large wound, PW post-wound, DP dermal papilla, AP alkaline phosphatase, FE follicular epithelium, HF hair follicle, Scale bars represent 500 µm (**a**), 10 μm (**d**, **e**)

We then sought to test whether Hh activation in dermal cells was capable of converting Wnt-active dermal fibroblasts to the DP fate. We induced forced Hh activation in Wnt responsive cells within small wounds, using the promoter of *Axin2*, a well-established target of canonical Wnt activity^59,60^ (*Axin2-CreER; R26-SmoM2*). TAM was administered into control and *Axin2-CreER; R26-SmoM2* from PW1d to PW30d. *Smo* overexpression in Wnt-active myofibroblasts resulted in extensive DP formation in small wounds (Fig. 6a–d). Genetic tracing of Axin2^+^ cells during HFN in reporter *Axin2-CreER; R26-Tomato* mice showed tomato expression in de novo DPs establishing that Axin2^+^ cells in wounds form DPs (Fig. 6e). In contrast, control wounds without Hh activation in Wnt-active dermal cells underwent scarring without HFN (Fig. 6a–d). These results show that Hh activation in Wnt-active dermal cells promotes their fate conversion into DP, the regenerative dermal niche for HF formation.

## Discussion

Our results demonstrate that wound repair can be redirected to promote regeneration following injury by modifying a key dermal signal (Fig. 6f). This study provides definitive evidence for a long-held concept that dermal cells are key components in determining wound healing outcome^8^. Our results suggest that the suppression of skin appendage regeneration in wound healing is due to the absence of dermal regeneration signals rather than intrinsic lack of regenerative competence in scarring cells. Installing developmental signals in the wound dermis may be a reasonable strategy to achieve regenerative healing in mammals. Epithelial activation of Wnt and Shh signaling was previously identified as critical for hair regeneration; however, these same pathways may also induce skin epithelial cancers when experimentally or pharmacologically augmented^61,62^. Our basic study with preclinical mouse models suggests that the capacity to create ectopic de novo DP in vivo by modulation of specific signals in the dermis may overcome this barrier and bring us closer to true skin renewal after injury.

Fibrotic scarring and epimorphic regeneration are frequently considered to be on opposite ends of the wound healing spectrum. This concept underpins attempts to promote regenerative healing by suppression of scarring mechanisms^8,63^. Our study suggests that scarring/fibrosis in skin wounds may not affect HF morphogenesis if the appropriate regenerative ques are applied. Although long-term activation of Wnt signaling, a hallmark of fibrotic repair, was observed in small wounds, the physiological level of Wnt signaling in scarring wounds did not negatively impact HFN in the presence of Shh activation. These studies provide evidence that fibrotic repair can be genetically subverted and offers possible tools to bypass extensive reprograming of adult skin cells into an “embryonic status” or for “stem cell transplantation” strategies to lead to regenerative healing in mammals.

HFN is largely a recapitulation of hair follicle development. However, the requirements for Shh regulation may be different in embryonic HF development and adult HFN. Shh-null mice can develop DP^16^ and Smo is dispensable for the initial establishment of DP in embryos^17^. Similarly, while epithelial Wnts are sufficient for embryonic HF development, both epithelial and dermal Wnts are needed for adult HFN^54,64^. The absolute requirement for Shh/Smo signaling in adult de novo DP formation may reflect a vulnerability in regeneration mechanisms compared to organogenesis in the embryo, whose development is often ensured by redundant compensatory mechanisms.

In conclusion, our study is the first to show that de novo DP can be created in adult skin by modulating a signaling pathway in the dermis. Future studies will be directed toward understanding the upstream mechanisms of Shh regulation, as well as downstream mechanisms leading to HF morphogenesis following de novo establishment of DP.

## Methods

### Mice

All animal protocols were approved by the Institutional Animal Care and Use Committee (IACUC) at NYU School of Medicine. All mice with proper genotype were used for the designed experiments regardless of sex. *LSL-Shh* mice in which mouse Shh is expressed under the control of β-actin promoter upon *Cre* medicated excision of stop sequence, were previously generated as described in Wang et al^23^. *Axin2-CreER*^59^ and *β-catenin* ^*fl(ex3)*^^65^ were obtained from indicated researchers. *Gli1-LacZ* (008211), *R26-SmoM2* (005130), *K14-CreER* (005107), *Shh* ^*fl/fl*^ (004293), *Pdgfra-CreER* (018280)*, Smo*^*fl/fl*^ (004526), *SM22-rtTA* (006875), *tetO-Cre* (006224), *Wls* ^*fl/fl*^ (012888), *Axin2-LacZ* (009120) and *R26-Tomato* reporter mouse (007908), were purchased from the Jackson laboratory.

To induce *CreER* activity, tamoxifen (TAM) treatment was performed by intraperitoneal injection (0.1 mg per g body weight) of a 20 mg per ml solution in corn oil^66^. To induce *rtTA* activity, mice were administered doxycycline-containing chow (20 g per kg, Bio-Serv).

### Wound experiment

Wound experiments were carried out with 3–4-week-old or 7–8-week-old mice as described in Ito et al.^11^. All wounding experiments were performed after anesthetization of mice with isoflurane. Briefly, for full-thickness large wound, 1 cm^2^ (1 X 1 cm) or 2.25 cm^2^ (1.5 X 1.5 cm) of skin were excised for 3–4-week or 7–8-week-old mice, respectively. For small wound, skin was excised by 4 mm full-thickness biopsy punch (Acuderm Inc.) as published^67^. For loss of function study of Hh pathway, wounds were harvested at PW21d by which the number of neogenic HFs was saturated in control wounds. For gain of function study of Hh pathway, samples were harvested around 30 days after wounding (PW30d~) to allow continuous formation of neogenic HFs.

### Whole-mount HFN assay

Whole-mount HFN assay to detect K17^+^ hair follicles and AP^+^ DP was performed as previously described^11^. To analyze hair follicle (dermal papilla and follicular epithelium) regeneration after wounding, wounded skin was harvested from the mice and incubated in 5 mM or 20 mM EDTA in PBS at 37 °C for 30 min ~2 h or overnight. The epidermis was gently separated from the dermis under a dissecting microscope (Axiovision Discovery V12, Zeiss, Germany). Both epidermis and dermis were fixed in 4% paraformaldehyde for 10 min at room temperature (RT) and rinsed with PBS. For the epidermis, standard DAB immunohistochemistry (see below) was performed with anti-K17 antibody (ab) (Abcam, 1:500). For the dermis, AP staining was performed. Dermis was incubated in NTMT solution (100 mM NaCl, 100 mM Tris-Cl (pH 9.5), 50 mM MgCl_2_, 0.1% Tween-20) for 10 min and then incubated in NTMT containing in NBT/BCIP (Roche, 1:50) solution at RT until color was visualized.

### Histochemistry

Immunohistochemistry was performed as published^66^. For paraffin sections, tissues were fixed in 4% PFA at 4 °C overnight and rinsed with PBS. Following sequential dehydration in ethanol and xylene, tissues were infiltrated by paraffin and embedded in fresh paraffin. The paraffin-embedded tissue blocks were chilled on ice for 10–15 min and cut at 6 µm thickness. The 6 µm tissue slices were flattened out on warm water and placed on microscope slides and dried out at 37 °C overnight. The tissue sections were rehydrated through xylene and graded series of ethanol (2 × 100%, 90%, 80%, 70%, and 50%) and rinsed with PBS. After antigen retrieval in Tris-EDTA (pH 8.0), the tissue sections were incubated with blocking solution (10% BCS in PBS containing 0.1% Tween-20) for 1 h at RT then appropriate primary antibodies at RT for 2 h or 4 °C overnight. After PBS washing, the tissue sections were incubated with secondary antibodies at RT for 1 h. If necessary, the second and third primary antibodies were used on the same tissue sections with corresponding secondary antibodies. Following PBS rinsing, the stained slides were mounted with mounting medium and stored at 4 °C for analysis. For frozen section, tissues were fixed in 4% PFA on ice for 10 min and embedded in OCT compound on dry ice. The frozen blocks were cut at 10 µm thickness, placed on microscope slides, and dried out at RT. After PBS washing, the tissue sections were incubated in blocking, primary, and secondary ab solutions as described above. All immunohistochemical analysis was observed and photographed on an upright Nikon Eclipse Ti or Zeiss Axiophot microscopes. Following antibodies were used: rabbit anti-K17 (1:500, Abcam), rabbit anti-Lef1 (1:100, Cell signaling), rabbit anti-Noggin (1:100, Abcam), rabbit anti-RFP (1:500, Rockland), rabbit anti-Shh (1:50, Santa Cruz), rabbit anti-SMA (1:100, Thermo Scientific), rabbit anti-F4/80 (1:100, Cell signaling), chicken anti-K14 (1:500, BioLegend), mouse anti-β-catenin (1:500, Sigma), mouse anti-SM22α (1:100, Abcam), mouse anti-AE13 (1:20, a gift of T.T. Sun), mouse anti-AE15 (1:20, a gift of T.T. Sun), mouse anti-K15 (1:100, NeoMarkers), and rat anti-CD34 (1:50, BD Bioscience). For histology, paraffin sections were stained with hematoxylin and eosin in accordance with a general method. To detect collagen protein, trichrome staining and picrosirius red staining were carried out using Masson’s Trichrome Stain Kit and Picrosirius Red Stain Kit, respectively (Polysciences). Trichrome staining was performed at NYUMC experimental pathology core.

### X-gal staining

X-gal staining was performed as published^68^. Skin wound tissues were fixed in 4% PFA at 4 °C for 30 min and rinsed with PBS. The tissues were incubated in X-gal rinse buffer (2 mM MgCl2, 0.01% Sodium deoxycholate, and 0.02% NP-40 in PBS) for 10 min at RT and then in X-gal (5-bromo-4-chloro-3-indolyl-β-d-galactopyranoside) staining buffer (0.7 mg/ml X-gal, 0.5 M K_4_Fe(CN)_6_ and 0.5 M K_3_Fe(CN)_6_ in X-gal rinse buffer) at RT or 37 °C until color was visualized. The tissues were photographed in whole mount using a dissection microscope (Zeiss, Discovery V12). The tissues were then dehydrated in standard graded series of ethanol, embedded in paraffin blocks and cut into 6-µm-thick sections. To visualize nucleus, the tissues were counterstained with nuclear fast red solution. Small wounds of *Axin2-LacZ* mice were incubated in 20 mM EDTA in PBS at 37 °C for 1 h to separate dermis from epidermis before X-gal staining.

### RNA-seq analysis

Skin wound tissues (PW11d) of control and *K14-CreER; LSL-Shh* mice were incubated in 20 mM EDTA solution at 37 °C for 30 min. After separation of epidermis from dermis, total RNA was isolated from epidermis and dermis using RNeasy Plus Micro-Kit (Qiagen) as described by the manufacturer. Total RNA was provided to Genome Technology Center at NYU Langone Medical Center for preparing RNA-seq libraries and sequencing. RNA-seq libraries were prepared using the Illumina TruSeq Stranded Total RNA library prep, after ribodepletion with Ribozero Gold kit (Illumina) starting from 200 ng of DNAse I treated total RNA, following the manufacturer’s protocol (15 cycles of PCR amplification). The amplified libraries were purified using AMPure beads, quantified by Qubit and QPCR, and visualized in an Agilent Bioanalyzer. The libraries were pooled equimolarly, and sequenced on two lanes of an Illumina HiSeq 2500 flow cell, v4 chemistry as paired-end 50. The differentially expressed genes (DEG) were submitted to DAVID for GO term analysis^69^. Top related enriched terms were selected and shown in the figures.

### Single-cell RNA-seq analysis

Skin wounds were collected from of both *SM22-rtTA; tetO-Cre; R26-SmoM2/Tomato* (*SM22-SmoM2*) and *SM22-rtTA; tetO-Cre; R26-Tomato* (control) mice 3 days after complete re-epithelialization (dox treatment from PW1d to PW12d) and incubated in 20 mM EDTA solution at 37 °C for 30 min to separate dermis from epidermis. The separated dermis was incubated in Dulbecco's Modified Eagle Medium (DMEM, Corning) containing 10% FBS (Corning) and 0.35% type I collagenase (Worthington) at 37 °C for 1 h. After rinsing with PBS, Tomato^+^ cells from isolated dermal cells were sorted with a FACSAria II cell sorter (BD biosciences). scRNA-seq libraries were prepared using the following: Single-Cell 3ʹ Reagent Kits v2: Chromium™ Single-Cell 3ʹ Library & Gel Bead Kit v2 PN-120237, Single-Cell 3ʹ Chip Kit v2 PN-120236, i7 Multiplex Kit PN-120262” (10x Genomics) 46 and the Single-Cell 3ʹ Reagent Kits v2 User Guide (Manual Part # CG00052 Rev A). Libraries were run on an Illumina HiSeq 4000 as 2 × 150 paired-end reads, one full lane per sample, for approximately > 90% sequencing saturation. Sequencing results were demultiplexed and converted to FASTQ format using Illumina bcl2fastq software. The Cell Ranger Single-Cell Software Suite (https://support.10xgenomics.com/single-cell-gene- expression/software/pipelines/latest/what-is-cell-ranger) was used to perform sample demultiplexing, barcode processing, and single-cell 3′ gene counting. The cDNA insert was aligned to the mm10/GRCm38 reference genome. Only confidently mapped, non-PCR duplicates with valid barcodes and UMIs were used to generate the gene-barcode matrix. Further analysis and visualization was performed using Seurat, an R package containing implementations of commonly used single-cell analytical techniques, including the identification of highly variable genes, dimensionality reduction, standard unsupervised clustering algorithms, and the discovery of differentially expressed genes and markers (http://www.ncbi.nlm.nih.gov/pubmed/26000488).

### Quantitative reverse transcription PCR (qRT-PCR)

Total RNA was isolated using RNeasy Micro-Kit (Qiagen) as described by manufacturer and reverse-transcribed with Superscript III First Strand Synthesis System (Invitrogen) for cDNA synthesis. cDNA was amplified using taqman probes and the ABI 7900HT SDS system. Transcripts were quantified relative to the housekeeping gene, GAPDH. The probes used for this study were listed in Supplementary Table 1.

### Hydroxyproline assay

To measure collagen content, hydroxyproline assay kit (Sigma) was used according to manufacturer’s protocol. Whole wound tissues were collected and homogenized in 100 µl of water per 10 mg tissue. After adding 100 µl of HCl (~12 M) per 10 mg tissue into the homogenized tissue, the mixture was hydrolyzed at 120 °C for 3 h. A total of 1–2 µl of supernatant was incubated in 100 µl of Chloramine T/Oxidation buffer mixture for 5 min and then, in 100 µl of diluted DMAB reagent for 90 min, sequentially. The absorbance was measured at 560 nm using SpectraMax M3 (Molecular Devices).

### Transmission electron microscopy

Transmission electron microscopy (TEM) was carried out in Microscopy Laboratory at NYU Langone Medical Center^70^. The harvested wounds were dissected (0.5 X1 cm) and put the wounds on top of paper tower. The skin wounds were fixed in the fixative containing 2.5% glutaraldehyde, and 2% paraformaldehyde in 0.1 M sodium cacodylate buffer (pH 7.2) with 1% tannic acid for 30 min and further dissected to 1 × 3 mm smaller pieces. Fixation process was continued in the same fixative at RT for 2 h, then 4 °C overnight. The skin then post-fixed with 1% osmium tetroxide for 2 h at RT, block staining in 1% uranyl acetate overnight at 4 °C, then dehydration in a standard manner and embedded in EMbed 812 (Electron Microscopy Sciences, Hatfield, PA) for transmission electron microscopy. Semi-thin sections were cut at 1 mm and stained with 1% Toluidine Blue to evaluate the orientation of the sample. Ultrathin sections (60 nm) were cut, mounted on copper grids and stained with uranyl acetate and lead citrate. Stained grids were examined under Philips cm-12 electron microscope (FEI; Eindhoven, The Netherlands) and photographed with a Gatan (4k X2.7 k) digital camera (Gatan, Inc., Pleasanton, CA).

### Statistical analysis and image processing

The whole-mount HFN assay was performed with at least three wound samples per genotype, and the data were representative of over three independent experiments. Data were represented as mean ± s.d. To calculate *p*-values, Student’s *t*-test was used on Microsoft Excel, with two-tailed tests and unequal variance. All graphs were generated by Microsoft Excel and GraphPad Prism. Images were processed using Image J and Adobe Photoshop. For transforming AP signals into colored dots using Photoshop, images of AP staining of wound were first turned into black and white, reduced background, and changed into red, green or cyan colors. Three images were then merged by overlapping the center of each wound.

## Electronic supplementary material

Supplementary Information

## Data Availability

RNA-seq data and scRNA-seq have been deposited in the Gene Expression Omnibus (GEO) database under accession codes GSE94893 and GSE112671, respectively. All other data of this study are available from the corresponding author upon reasonable request.
