# Supplementary material for: Hedgehog stimulates hair follicle neogenesis by creating inductive dermis during murine skin wound healing
Source: Nat Commun. 2018 Nov 21;9:4903. doi: 10.1038/s41467-018-07142-9 (PMC6249328; doi:10.1038/s41467-018-07142-9)
Supplement: Supplementary file 1 — Supplementary Information [file 41467_2018_7142_MOESM1_ESM.pdf]

Supplementary Information

Hedgehog stimulates hair follicle neogenesis by creating inductive dermis during murine skin wound healing

Chae Ho Lim et al.

Supplementary Figures and Table

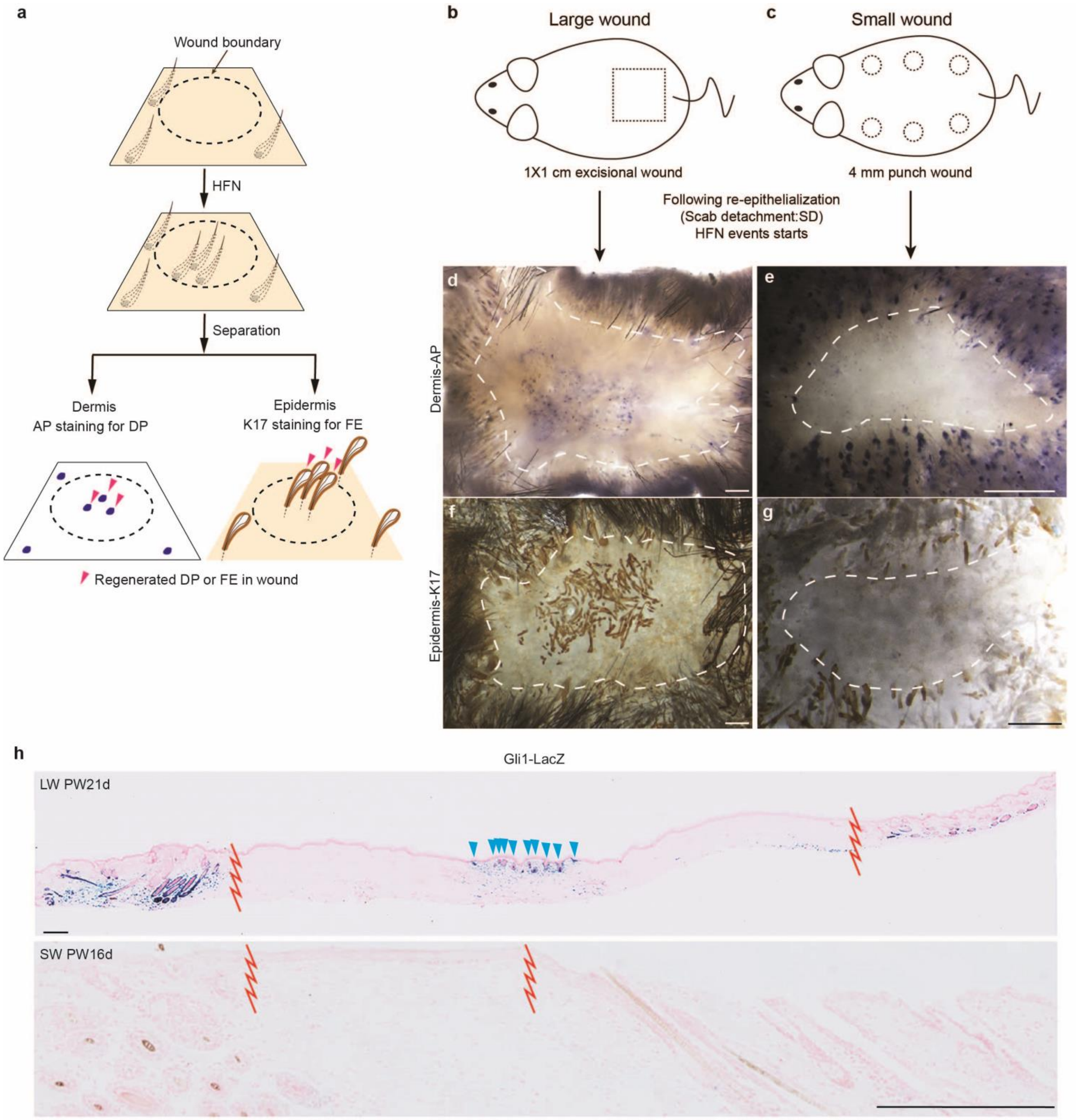

Supplementary Figure 1. Large wounds induce Hh signaling and hair follicle neogenesis (HFN) unlike small wounds

a, Schematic illustration of whole mount HFN assay. **b and c,** Schematic illustrations of full-thickness large wound (LW) and small wound (SW), respectively. **d and e,** Whole mount AP staining on wound dermis of wild-type mice. LW (d) and SW (e). **f and g,** Whole mount K17 staining on wound epidermis of wild-type mice. LW (f) and SW (g). Wounds were collected at PW21d for large wounds and at PW30d for small wounds. **h,** Gli1-LacZ signals on tissue section at PW21d (LW) and PW16d (SW). Arrowheads show regenerated HFs. Dashed circle: wound boundary. AP: alkaline phosphatase, DP: dermal papilla, FE: follicular epithelium, PW: post-wound. Scale bars represent 500 μm (d-h).

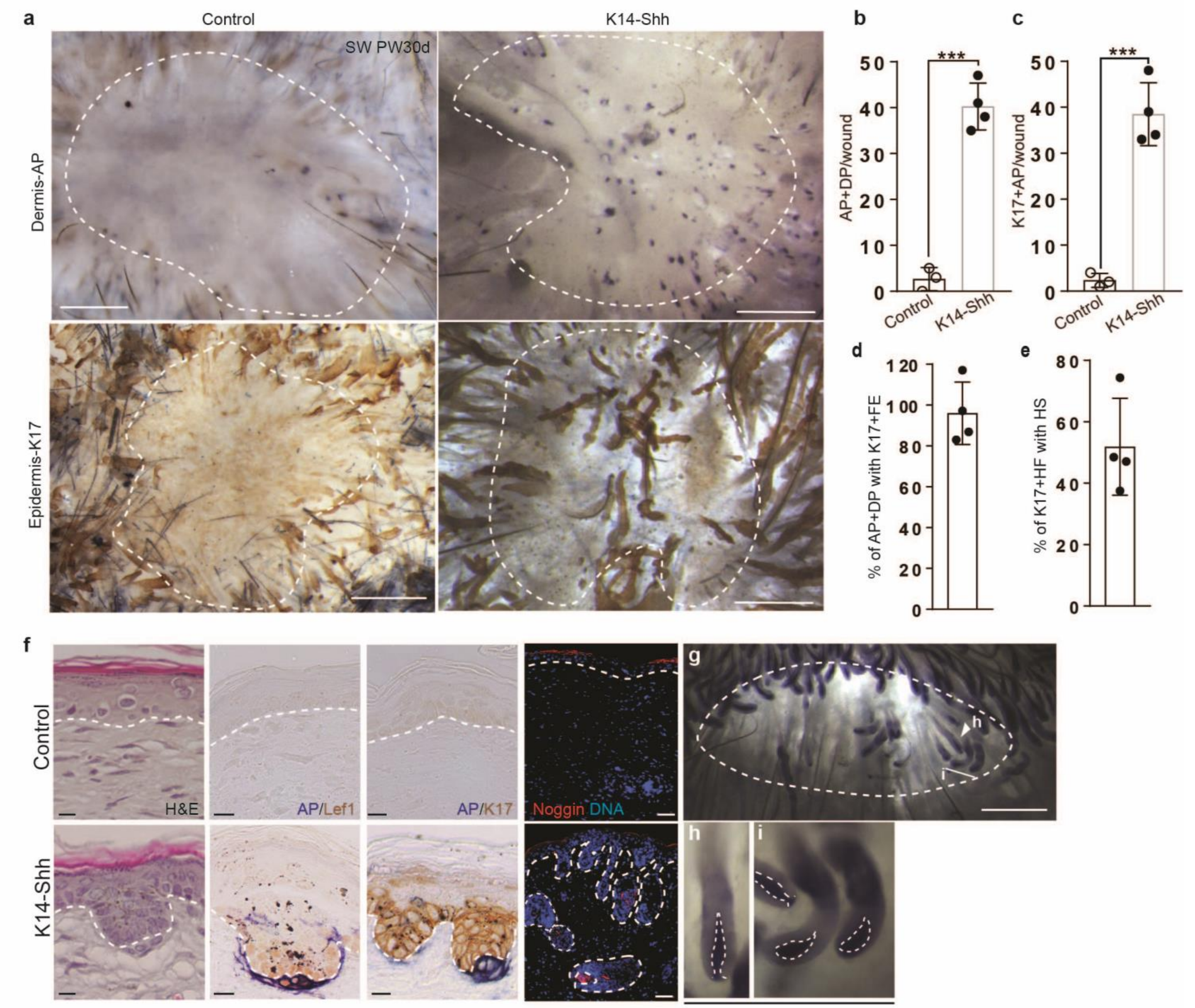

Supplementary Figure 2. Sufficiency of epithelial Shh to produce de novo hair follicles in non-regenerative wound

a-i, *K14-CreER; LSL-Shh (K14-Shh)* and littermate controls were subjected to SW and treated with TAM from PW1d until tissue harvest at PW30d (n=18W (7-12M) per condition). Whole mount HFN assay (a) and quantifications (b and c). Percentage of AP^+ DP with $K17^+$ FE (d). Percentage of $K17^+$ HF with hair shaft (HS) (e). H&E and immunohistochemical analyses with indicated markers (f). Side view of AP staining of whole mount wound skin of *K14-Shh* (g). Magnified views of regenerated HFs in wound. Dashed white circles represent DP. (h and i). n: number of wounds (W) or mice (M), Data are represented as mean ± s.d., ***$p<0.001$; Student's t-test, Dashed white circle: wound boundary, Dashed line: epidermis-dermis border, SW: small wound, PW: post-wound, DP: dermal papilla, AP: alkaline phosphatase, FE: follicular epithelium, HF: hair follicle, HS: hair shaft, Scale bars represent 500 µm (a, g), 50 µm (one column on the right in f), 10 µm (three columns on the left in f).

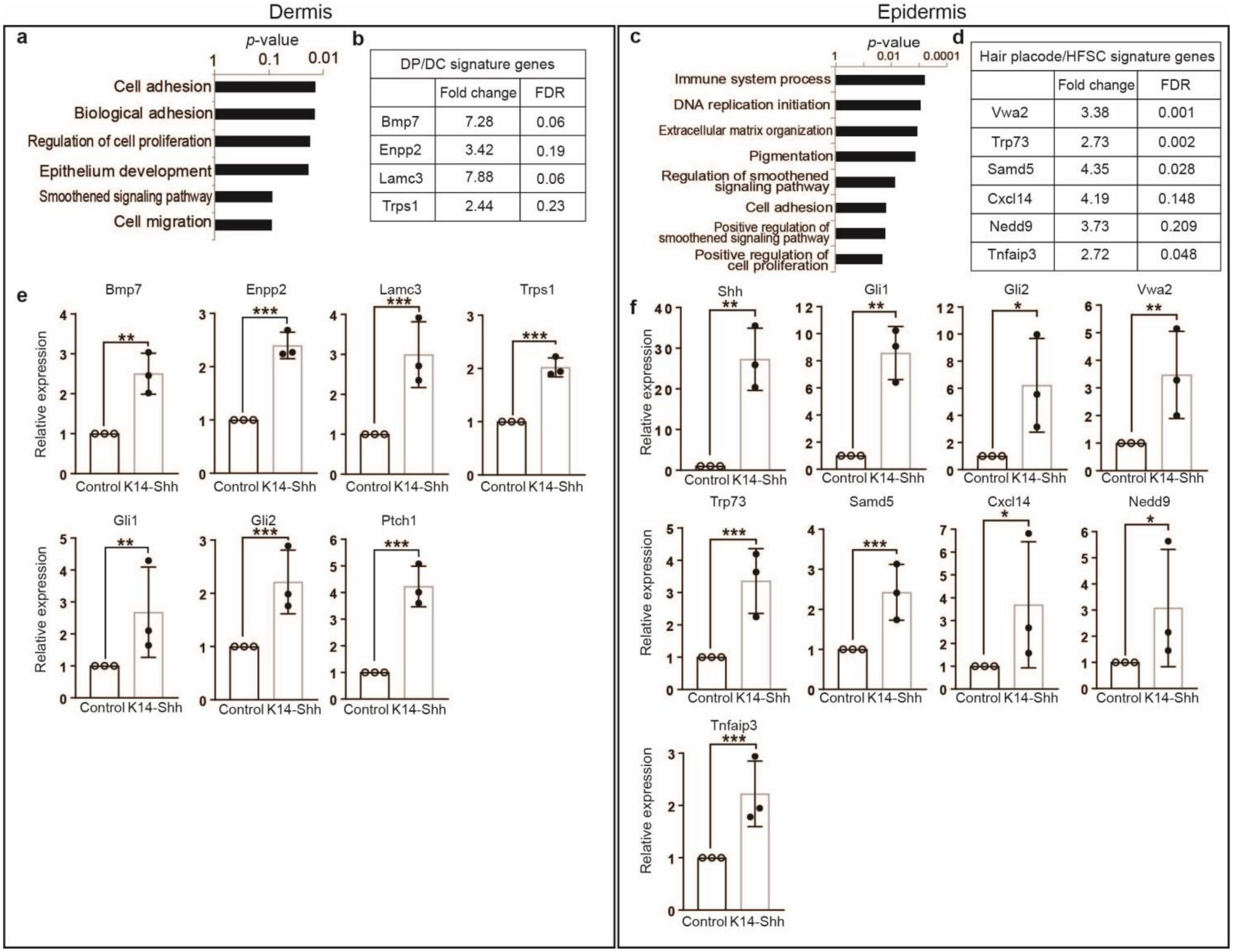

Supplementary Figure 3. Epithelial Shh overexpression in adult wounds leads to up-regulation of signature genes for embryonic HF morphogenesis

a-f, *K14-CreER; LSL-Shh* (*K14-Shh*) mice and littermate controls were subjected to small wound (SW) and treated with TAM from PW1d until tissue harvest at PW11d (n=12W (4M) per condition). Gene ontology (GO) analysis of differentially expressed genes (DEGs) (a and c). DP and dermal condensate (DC) signature genes upregulated in dermis of *K14-Shh* (b). Hair placode and hair follicle stem cell (HFSC) signature genes upregulated in epidermis of *K14-Shh* (d). qRT-PCR with indicated genes (e and f). n: number of wounds (W) or mice (M), Data are represented as mean ± s.d., $^{*}p<0.05$; $^{**}p<0.01$; $^{***}p<0.001$; Student's t-test, PW: post-wound.

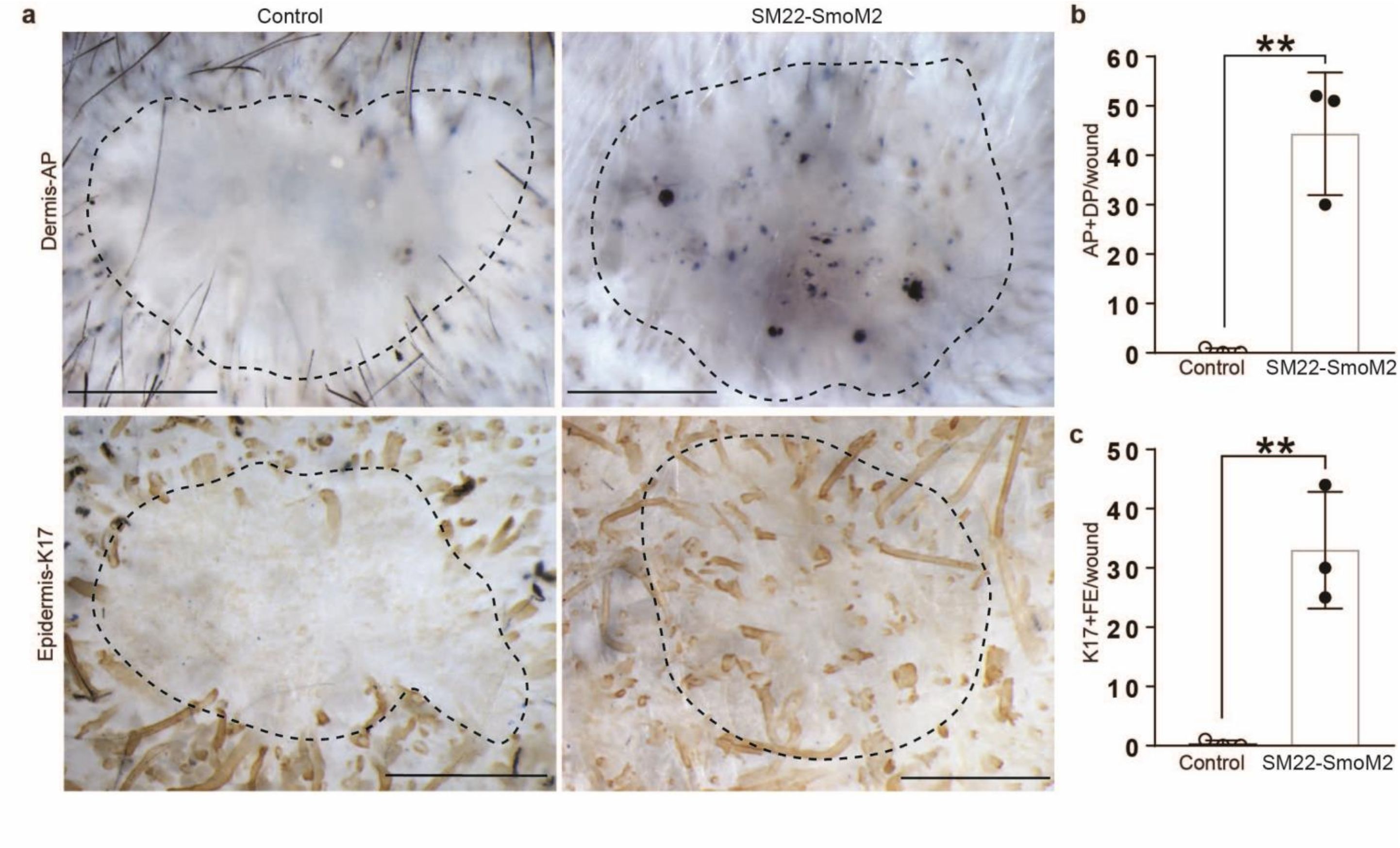

Supplementary Figure 4. Dermal Hh activation after complete re-epithelialization is sufficient to promote HFN in non-regenerative small wound

a-c, *SM22-rtTA; tetO-Cre; R26-SmoM2* (*SM22-SmoM2)* and littermate controls were subjected to small wound and treated with doxycycline from scab detachment (PW10 ± 2d) until tissue harvest at PW 46d (n=15W (3-4M) per condition). Whole mount HFN assay (a) and quantifications (b and c). n: number of wounds (W) or mice (M), Data are represented as mean ± s.d., **$p<0.01$; Student's t-test, Dashed circle: wound boundary, DP: dermal papilla, AP: alkaline phosphatase, FE: follicular epithelium, PW: post-wound, Scale bars represent 500 μm.

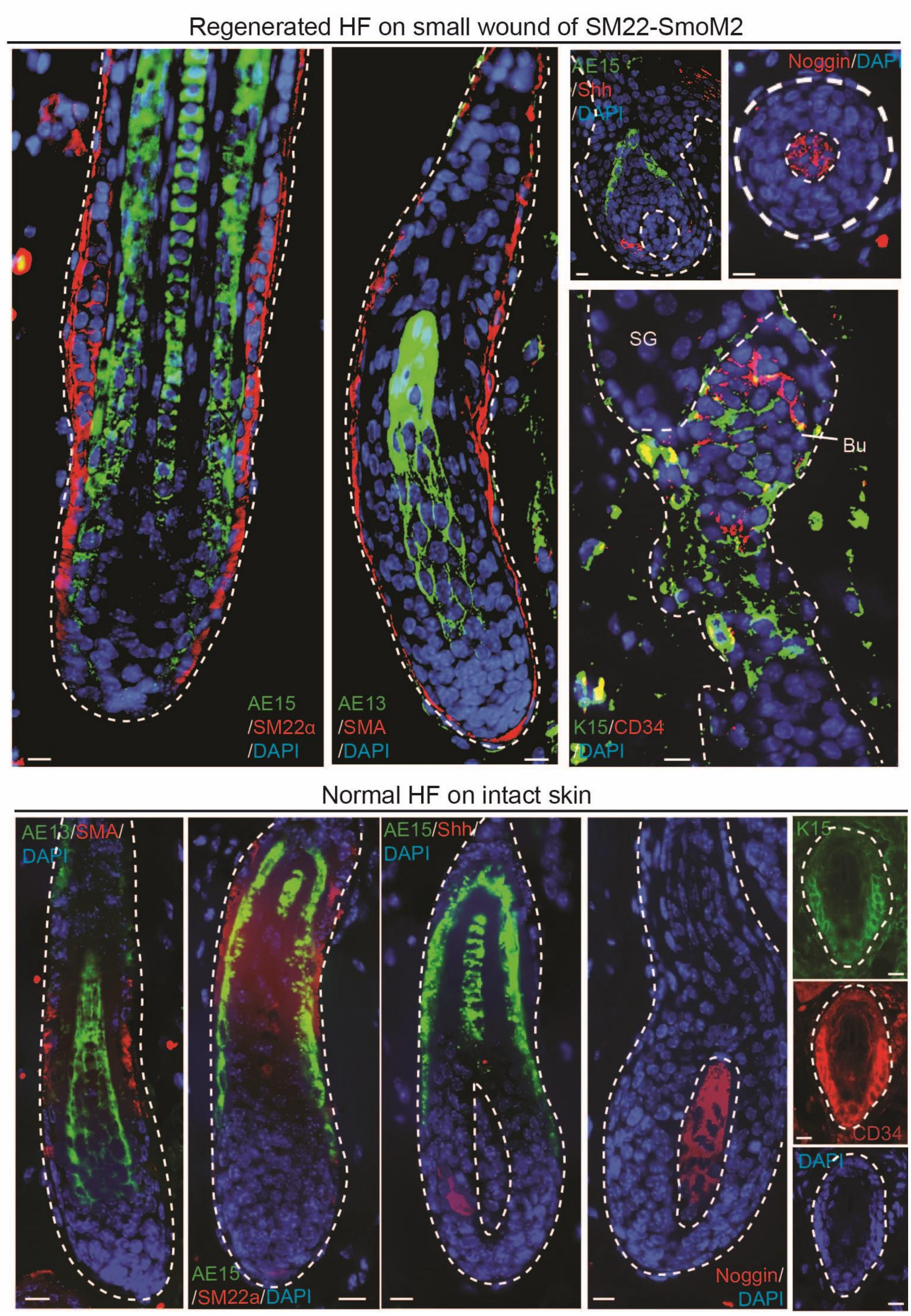

Supplementary Figure 5. Hh-driven regenerated HF resemble normal HF
Immunofluorescence with indicated markers on neogenic hair follicles (HFs) in *SM22-rtTA; tetO-Cre: R26-SmoM2* (*SM22-SmoM2*) mice at PW 34d. Normal HFs in intact skin of wild-type mice were analyzed as control. Dashed line: epidermis-dermis border, Bu: bulge stem cell area, SG: sebaceous gland, Scale bars represent 10 µm.

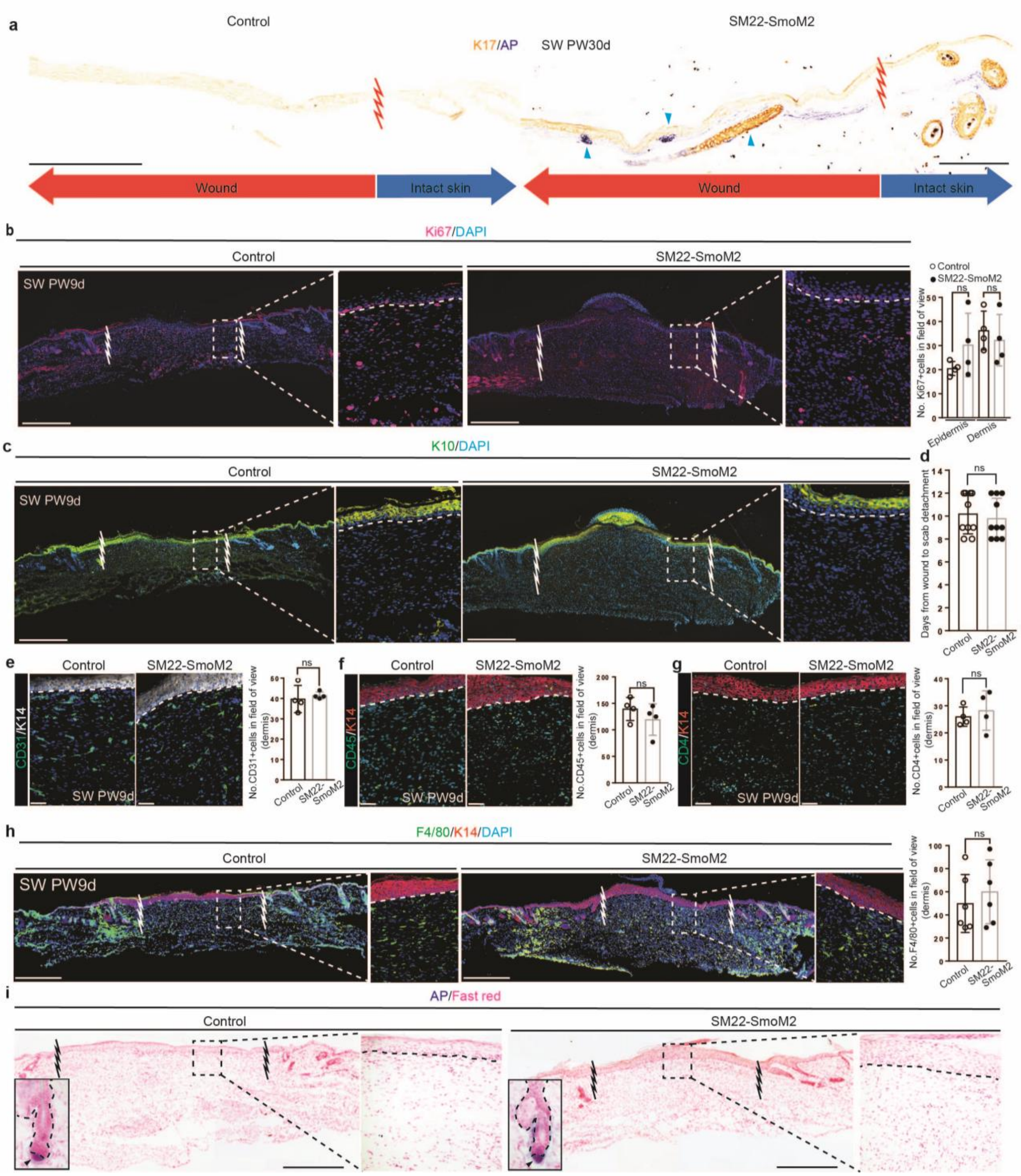

Supplementary Figure 6. Dermal Hh activation does not alter proliferation, differentiation, angiogenesis and immune responses in small wound

a, AP/K17 staining on small wound (SW) of *SM22-rtTA; tetO-Cre; R26-SmoM2* (*SM22-SmoM2)* and littermate controls at PW30d. Arrowheads show regenerated HFs. **b-i**, *SM22-SmoM2* and littermate controls were subjected to SW and treated with doxycycline from PW1d until tissue harvest at PW9d (n=2W (2M) per condition). Immunohistochemical analyses for indicated markers to assess proliferation (b), epidermal differentiation (c), endothelial cell distribution (e), immune cell infiltration (f, g), macrophage distribution (h) and AP activity (i). Insets show HFs including AP$^+$ DP in intact skin (i). Days from wound to scab detachment (d). n: number of wounds (W) or mice (M), Data are represented as mean ± s.d., ns: non-significant; Student's t-test, Dashed line: epidermis-dermis border, PW: post-wound, Scale bars represent 500 µm (a-c, h, i), 50 µm (e-g).

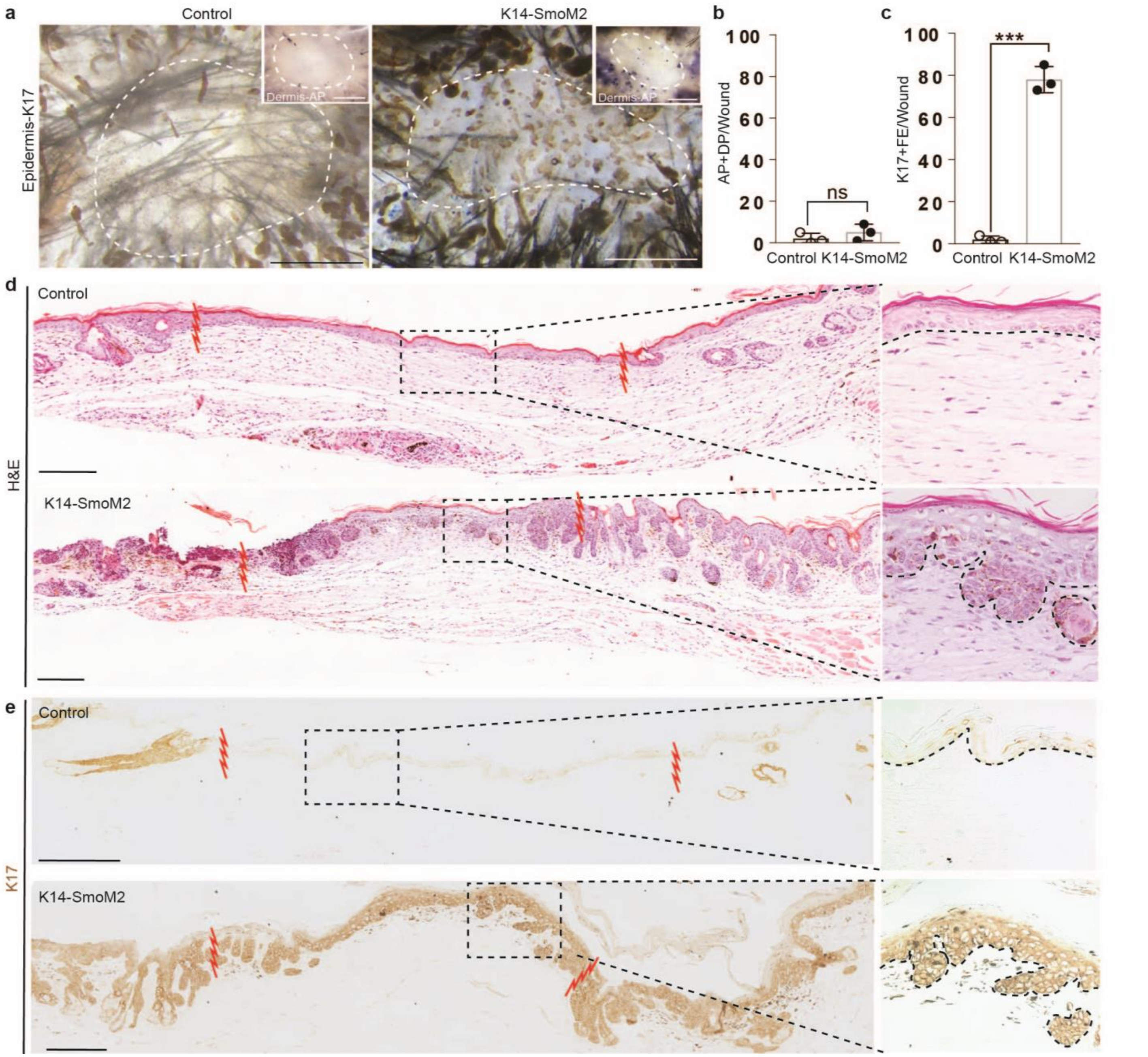

Supplementary Figure 7. Epidermal Smo activation does not promote dermal papilla formation following wounding

a-e, *K14-CreER; R26-SmoM2* (*K14-SmoM2*) and littermate controls were subjected to small wound and treated with TAM from PW1d until tissue harvest at PW30d (n=9W (4-5M) per condition). Whole mount HFN assay (a) and quantifications (b and c). Insets show the corresponding whole mount AP staining in wound dermis. H&E (d) and K17 staining (e) on sections. n: number of wounds (W) or mice (M), Data are represented as mean ± s.d., ns: non-significant; ***p<0.001; Student's t-test, Zigzag line and dashed white circle: wound boundary, Black dashed line: epidermis-dermis border, DP: dermal papilla, AP: alkaline phosphatase, FE: follicular epithelium, PW: post-wound, Scale bars represent 500 µm (a), 100 µm (d, e).

Gene	Assay ID
Bmp7	Mm00432102_m1
Enpp2	Mm00516572_m1
Lamc3	Mm01324510_m1
Trps1	Mm00459612_m1
Vwa2	Mm01192757_m1
Trp73	Mm01261128_m1
Samd5	Mm01192757_m1
Cxcl14	Mm00444699_m1
Nedd9	Mm01324843_m1
Tnfaip3	Mm00437121_m1
Gli1	Mm00494654_m1
Gli2	Mm01293111_m1
Shh	Mm00436528_m1
Ptch1	Mm00436026_m1
GAPDH	Mm99999915_g1

Supplementary Table 1. A list of taqman probes used for qRT-PCR
